# White Rot Fungi (*Hymenochaetales*) and Esca of Grapevine: Insights from Recent Microbiome Studies

**DOI:** 10.3390/jof7090770

**Published:** 2021-09-17

**Authors:** Giovanni Del Frari, Helena Oliveira, Ricardo Boavida Ferreira

**Affiliations:** LEAF—Linking Landscape, Environment, Agriculture and Food—Research Center, Instituto Superior de Agronomia, Universidade de Lisboa, 1349-017 Lisbon, Portugal; heloliveira@isa.ulisboa.pt (H.O.); rbferreira@isa.ulisboa.pt (R.B.F.)

**Keywords:** *Fomitiporia mediterranea*, grapevine leaf stripe disease, grapevine trunk diseases, *Phaeomoniella chlamydospora*, *Phaeoacremonium*, interveinal necrosis

## Abstract

Esca is a major grapevine trunk disease that heavily affects vineyards in the Northern hemisphere. The etiology and epidemiology of this disease have been subject of dispute ever since the earliest disease reports. The reason behind such debate is the presence of multiple internal and external symptoms, as well as several putative and confirmed wood pathogens. While the role of pathogenic fungi, as causal agents of wood symptoms, has been thoroughly assessed, their role in the expression of leaf symptoms remains to be fully elucidated. In this review, we analyzed etiological and epidemiological data, with a special focus on the microbiological aspect of esca and the involvement of *Hymenochaetales* (Basidiomycota). Vineyard studies have associated leaf symptoms with the presence of white rot, most frequently caused by *Fomitiporia mediterranea* (*Hymenochaetales*), while tracheomycotic fungi are commonly found, with similar abundance, in symptomatic and asymptomatic vines. Pathogenicity trials have excluded a direct effect of *Hymenochaetales* species in triggering leaf symptoms, while the data concerning the role of tracheomycotic fungi remains controversial. Recent microbiome studies confirmed that *F. mediterranea* is more abundant in leaf-symptomatic vines, and treatments that effectively control leaf symptoms, such as sodium arsenite spray and trunk surgery, act directly on the abundance of *F. mediterranea* or on the presence of white rot. This suggest that the simultaneous presence of *Hymenochaetales* and tracheomycotic fungi is a pre-requisite for leaf symptoms; however, the relation among fungal pathogens, grapevine and other biotic and abiotic factors needs further investigation.

## 1. Introduction

**Figure 1:**
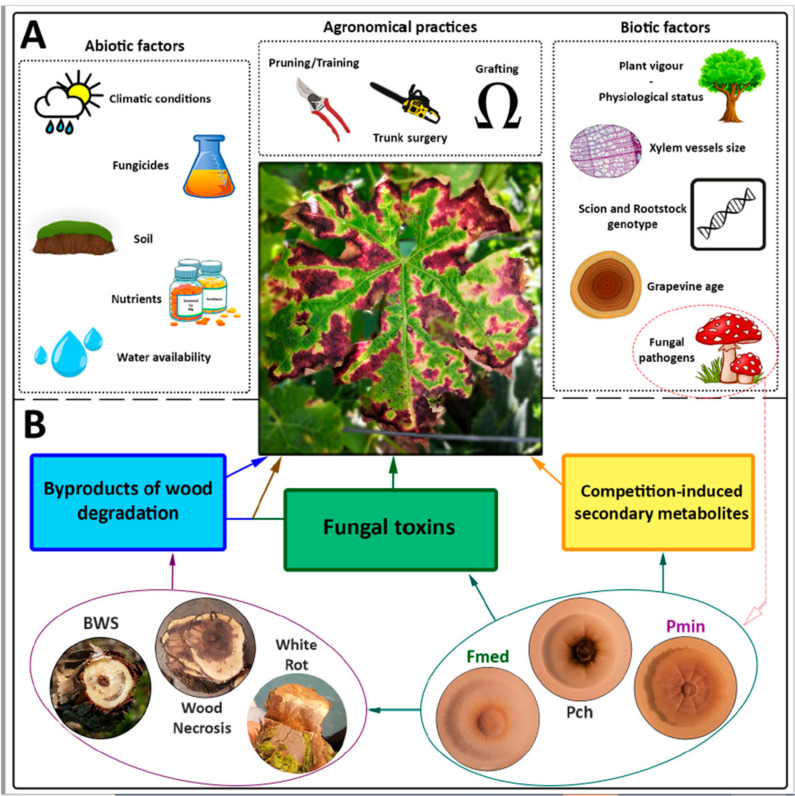
Agronomical practices and biotic and abiotic factors known to influence leaves’ expression of esca symptoms in leaves (**A**). Three main hypotheses for leaf symptoms triggering fungal pathogens and related symptoms in the wood (**B**). BWS: brown wood streaking, Fmed: *Fomitiporia mediterranea*, Pch: *Phaeomoniella chlamydospora*, Pmin: *Phaeoacremonium minimum*. References for (**A**): [10,11,13]. References for (**B**): [19]. Open source images in (**A**) were retrieved and adapted from pixabay.com.

Since the early 2000′s, extensive scientific research has deepened our understanding of the leaf symptoms associated with the esca disease in grapevine. Despite significant efforts in the study of leaves and wood biochemistry, anatomy, microbiology, physiology and transcriptomics [1,2,3,4,5,6,7,8], researchers were not able to pinpoint a single, primary cause for leaves’ manifestation of symptoms. Under current classification [9], esca symptoms in leaves, also known as tiger stripes, occur in vines affected by grapevine leaf stripe disease (GLSD) and esca proper, and they are known to be correlated to agronomical practices (e.g., pruning systems, grafting) and other biotic and abiotic factors [10,11,12,13,14] (Figure 1A). The involvement of multiple factors complicates attaining a clear etiological pattern, which has prevented scientists from identifying a reliable means of control. Nevertheless, three control strategies have proven effective in reducing leaf symptoms’ manifestation, namely (i) spraying vines with sodium arsenite (today, banned from vineyards) [15]; (ii) spraying the grapevine canopy with a mixture of seaweed and minerals [16,17]; and (iii) trunk surgery, also known as curettage or ‘metodo Armano’ (in North-East Italy; [18]). However, the modes of action of all three techniques remain to be fully clarified.

**Figure 2:**
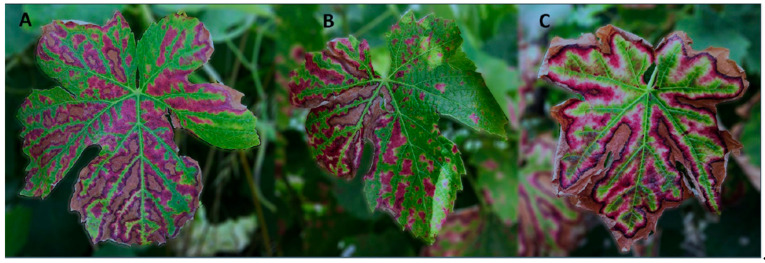
Leaves of *Vitis vinifera* cv. Touriga Nacional affected by interveinal necrosis (Lisbon, June 2021). Necrotic spots may occur on the whole leaf lamina (**A**) or parts of it (**B**), occasionally involving leaf margins (**C**).

Details over the past and modern views of esca and leaf symptoms were examined by several authors, including [6,9,13,19,20], while the involvement of white rot fungi (*Hymenochaetales*) in grapevine trunk diseases has been reviewed by [21,22,23]. Mugnai et al. [19] provided a detailed description of leaf symptoms, since their onset to the advanced stages, which we quote: symptoms on leaves consist of light green or chlorotic, rounded or irregular spots between the veins or along the leaf margins that usually spread outward to the distal parts of the shoots. The spots, initially small and scattered over the lamina, gradually expand and coalesce, become partly necrotic, and ultimately leave only a narrow strip of unaffected green tissue along the main veins. As the chlorotic tissue turns yellow-brown or red-brown, the diseased leaves assume a “tiger-stripes” pattern. Sometimes the necrotic areas of the lamina dry out and become detached, leaving irregular leaf margins. Other symptoms, such as clearing, puckering, glistening, and distortion of the leaf lamina, are less common” [19]. Leaf symptom patterns often vary, and necrotic areas may involve interveinal tissues in the whole leaf or in only part of it, with or without affecting the leaf’s margins (Figure 2). Today, tiger stripes are encountered in all vine-growing regions of the northern hemisphere [24], in South Africa and Australia they are infrequent [25,26], while no tiger stripes were reported in South America and New Zealand [27,28,29,30].

In their review, Mugnai et al. [19] list three main hypotheses for leaf symptoms’ expression. The ‘toxins hypothesis’, in which fungal toxins are translocated from perennial wood to leaves, causing phytotoxicity; the ‘byproducts of wood degradation hypothesis’, similar to the toxins hypothesis, but wherein phytotoxicity is caused by molecules that derive from the breakdown of woody tissue; and a combination of both hypotheses (Figure 1B). Over the following years, new theories have been proposed [31,32], but they still need to be further explored. While the toxins hypothesis holds as the most widely accepted, due to the partial success in reproducing tiger stripes-like symptoms on detached leaves [33], numerous points remain to be addressed. If the toxins theory is correct, what is the role of agronomical practices and abiotic factors on symptoms’ manifestation? What are the modes of action of effective control strategies (e.g., trunk surgery)? Why did scientists hardly succeed in reproducing leaf symptoms *in planta,* by artificial inoculations with toxin-producing fungal pathogens (e.g., *Phaeomoniella chlamydospora* and *Phaeoacremonium* spp.)?

Over the last three years, four microbiome studies allowed us to widen our understanding of the role of fungal communities in the manifestation of tiger stripes. In this article, we will focus on the microbiological aspect of esca symptoms in leaves, in particular, on the role of white rot fungi, and we will examine both past knowledge and the main findings of these recent studies, discussing the implications over leaf symptoms’ etiology.

## 2. Natural Infections in the Field

Numerous researchers investigated the fungal wood microbiota in plants manifesting tiger stripes and compared it to asymptomatic vines, employing both culture-dependent and culture-independent approaches. On the contrary, few studies devoted their attention to healthy vineyards that never manifested tiger stipes [34,35,36,37]. Interestingly, these authors did not report the presence of white rot fungi in the endophytic community of their examined plants.

In a majority of available studies, and as described in early esca research [38], leaf-symptomatic vineyards are affected by white rot most often associated with *Fomitiporia mediterranea* (Fmed) and by symptoms in wood, caused by tracheomycotic fungi (e.g., brown wood streaking, necrosis), most frequently *P. chlamydospora* (Pch) and/or *Phaeoacremonium minimum* (Pmin). Members of the *Botryosphaeriaceae* are also frequently isolated in esca-related studies, despite their role remaining to be fully clarified. According to the classification by Surico (2009) [9], vines affected by the above-mentioned symptoms fall in the category ‘esca proper’. On the other hand, studies where the presence of tiger stripes was observed in vines affected by wood symptoms caused by tracheomycotic fungi but not white rot (grapevine leaf stripe disease; [9]) remain few. To the best of our knowledge, in literature, there are no field studies describing vineyards affected exclusively by white rot, and not by wood symptoms such as brown wood-streaking and/or necrosis, regardless of the presence of tiger stripes in leaves (white rot syndrome, also known as ‘esca’; [9,39]).

### 2.1. Esca Proper

In Spain, Fmed was more frequently isolated from vines showing esca symptoms in leaves than other wood pathogens, such as Pch or Pmin, while vines showing decline but not leaf symptoms had higher frequencies of Pmin and Pch than Fmed [40]. Elena et al. [41] reported that Fmed was isolated exclusively from white rot or necrotic tissues of grapevines showing leaf symptoms, whereas Pch was isolated from both symptomatic and asymptomatic vines, and Pmin was more frequently isolated from asymptomatic ones. 

In Veneto (Italy), Fmed was isolated from 75% of vines showing typical tiger-stripe foliar symptoms and never from asymptomatic ones [42]. Moreover, Pch and Pmin were isolated in similar abundances in both symptomatic and asymptomatic vines [39]. In Apulia, Pollastro et al. [43] describes white rot as the most common wood alteration in vines exhibiting leaf symptoms. 

In Germany, Fischer and Kassemeyer (2003) [44] concluded that ‘wood decay caused by Fmed seems to be the main reason for esca disease in the geographic area under study’, with a frequency of isolation of 63%, from symptomatic plants (Pch and Pmin, 30%). 

In France, Larignon and Dubos (1997) [45] isolated, in nearly all leaf-symptomatic vines (*n* = 309) Fmed, along with Pch and Pmin. Fmed was isolated from white rot tissue but also from other types of necrosis. Peros et al. [46] examined 210 vines with foliar symptoms, revealing that 91.4% presented white rot, associated in most cases with Fmed. Vines were always colonized also by other wood pathogens (e.g., Pch and/or Pmin). The authors concluded that “interveinal necrosis was more often linked with the presence of white rot due to *F. mediterranea*”. Kuntzmann et al. [47] reported a high frequency of isolation of Fmed and *Stereum hirsutum* in leaf-symptomatic plants, along with other Ascomycetes, such as Pch and *Diplodia seriata*. In turn, Bruez et al. [48] identified white rot in all leaf-symptomatic vines, but not in asymptomatic ones. Pch and Pmin were encountered exclusively in a single season, at low abundances, while *Botryosphaeriaceae* were predominant. Ouadi et al. [49] described that the largest amount of white rot necrotic wood was found in the trunk and cordons of grapevines that expressed esca foliar symptoms. This observation is supported by a previous study, wherein a logistical model indicated that white rot in the cordons was the best predictor for the chronic form of esca [50]. In fact, the amount of white rot significantly increased the probability of chronic symptoms expression. 

In Portugal, in a study carried out in the Dão Region, Fmed was isolated from 83% of the vines showing leaf symptoms of esca and white rot. In the remaining plants, the authors occasionally found an incipient presence of spongy white decay, although Fmed was not isolated. Pch, *Phaeoacremonium* spp. and *Botryosphaeria* spp. were among the most abundant fungi isolated, depending on the woody tissue examined [51]. 

In South Africa, White et al. [26,52] described esca-diseased vines as having brown-black internal discoloration accompanied by white rot. In addition to Pch and Pmin, the authors identify ten Basidiomycetes. Unfortunately, the authors did not specifically discern between leaf symptoms and wood symptoms when describing esca-diseased vines.

### 2.2. Grapevine Leaf Stripe Disease

Among the few studies where tiger stripes were observed in the (putative) absence of white rot, and/or white rot-associated fungi, we find that of Edwards et al. [53], in Australia. The authors reported that white rot Basidiomycetes were rarely found, and leaf symptoms were associated mostly with Pch and Pmin, even though these pathogens were also isolated in asymptomatic vines (symptomatic vines examined, *n* = 7). In a study by Calzarano and Di Marco (2007) [38], the authors detected white rot in over 90% of leaf-symptomatic vines (vineyard 1), while this percentage reached ~50% in vineyard 2. Since the remaining vines were only affected by wood discoloration, the authors concluded that white rot is not a prerequisite for tiger stripes’ manifestation. Romanazzi et al. [54] detected low abundances (in mature vineyards) or absence (in young vineyards) of Fmed in esca leaf-symptomatic plants. Hofstetter et al. [55] examined 38 leaf-symptomatic adult vines, using a non-destructive wood sampling method, and Fmed was isolated infrequently (5% of inspected vines), while both Pch and *Phaeoacremonium* spp. where isolated in similar abundances in leaf-symptomatic and asymptomatic vines. In a recent study, Raimondo et al. [56] examined vines (*n* = 138) showing GTD-related symptoms, including tiger stripes, from several vineyards, without reporting the presence of a single white rot-associated fungus. 

Biases in cultural methods for the isolation of endophytic Basidiomycetes are well known [46,57], and they may have contributed to an underestimation of the presence and abundance of *Hymenochaetales* in esca research. Moreover, studies in which endophytic fungi were isolated exclusively from healthy wood or wood affected by brown streaking (e.g., [53]), certainly missed the presence of Fmed. In fact, this fungus colonizes primarily white rot tissue, the necrotic wood surrounding white rot and, occasionally, central or sectorial necroses [19,44,45,51]. For this reason, it is fundamental to examine thoroughly the entirety of vine trunks and cordons for the presence of white rot, with a special focus in the area near pruning wounds, where white rot is thought to originate [19], not to miss the woody tissue where Fmed is most frequently isolated from. In some GLSD studies, the authors either did not report the way that vines were sectioned and inspected for the presence of white rot [54,56], or used a non-destructive sampling approach [55], which prevented an accurate assessment of the wood symptomatology.

In addition to the biases mentioned above, it is critical to address the ‘vine age’ factor when studying the etiology and epidemiology of esca. In fact, sampling is often done, and more easily accepted by the growers, on adult vineyards, which have a higher probability of being affected by white rot, when compared to young ones, as this symptom develops slowly. For this reason, there is an unbalanced access to equal proportions leaf-symptomatic vineyards of different ages, which may have led to under- or overestimate the presence (or lack) of white rot [58].

To summarize, available evidence suggests that (i) vineyards that never manifested tiger stripe leaf symptoms lack the presence of white rot-associated fungi; (ii) in the majority of cases, vineyards manifesting tiger stripes are affected by white rot and colonized by white rot fungi, even if the presence of white rot does not necessarily lead to leaf symptoms; (iii) leaf-symptomatic vineyards are always colonized by pathogenic Ascomycetes (e.g., Pch, *Phaeoacremonium* spp., *Botryospaheriaceae*), in addition to white rot fungi. 

## 3. Fulfilling Koch’s Postulates

### 3.1. Tracheomycotic Fungi

Over the last 20 years, numerous pathogenicity studies successfully replicated wood symptoms, the most common being brown wood streaking and necrotic lesions, by artificial inoculations with Pch, *Phaeoacremonium* spp. and other Ascomycetes, both under greenhouse and field conditions. Despite this, only few of them reported esca foliar symptoms manifesting in inoculated vines, either months or years post-inoculation. In an experiment by Sparapano et al. [59], Pch and Pmin, inoculated alone or in combination, contributed to the expression of tiger stripes in 0–38.8% and 0–25.9% of inoculated plants, of cvs. Italia and Matilde respectively. Similar results were obtained by Feliciano et al. [60], who observed leaf symptoms in 0–24% of vines inoculated with Pch or *Phaeoacremonium* spp., depending on vine cultivar. Also Úrbez-Torres et al. [61] tested Pch and *Phaeoacremonium* spp., in cv. Baco Noir, observing tiger stripes. However, the authors do not specify the percentage of leaf-symptomatic plants. Recently, Ye et al. [62] replicated leaf symptoms in 30% of inoculated plants using Pmin. 

These results suggest that Pch and some species of *Phaeoacremonium* play a role in the expression of leaf symptoms; however, the number of studies wherein such symptoms were not replicated far outweighs those in which it did (e.g., [19,45,63]). Therefore, the mere presence of these fungi in (symptomatic) wood seems necessary, albeit insufficient, to trigger tiger-stripes expression. Some questions naturally arise, for example (i) why are leaf symptoms reproduced in such low percentages? (ii) Why do some cultivars seem to be unaffected, at the leaf level, by artificial inoculations? (iii) If multiple fungi/bacteria need to be simultaneously present to trigger leaf symptoms, what is the role of the resident endophytic microbiome? 

In conclusion, while Koch’s postulates were certainly fulfilled as concerns wood symptoms, the conflicting evidence suggests that other factors play a key role in the etiology of leaf symptoms, leaving Koch’s postulates at least in part unfulfilled.

### 3.2. Hymenochaetales

The role of Fmed and other *Hymenochaetales*, in the development of wood and foliar symptoms in grapevines, was also object of investigation. Researchers aimed to replicate both the internal and external symptoms that are frequently observed in esca-affected plants, and aimed, therefore, at fulfilling Koch’s postulates. In literature, we encountered 11 studies, which examined 13 grapevine cultivars, 10 *Hymenochaetales* species and 20 Fmed isolates. They were conducted either under greenhouse or field conditions, in experiments that lasted between 3 and 96 months (Table 1). 

A majority of the studies conducted to date, both in potted young and adult plants (1–10 years old), describe the development of wood symptoms, such as discoloration, brown streaking, brown lesions and necrosis. Yet, the typical white rot symptom has been successfully reproduced exclusively in adult plants (5–13 years old). Regarding the replication of tiger stripes, only three studies report the appearance of ‘esca or esca-like’ leaf symptoms. However, such ‘esca-like’ symptom, described in the study by Sparapano et al. [59] as ‘chlorosis and reddening of the leaf margins; necrosis of large parts of the lamina’, does not fully correspond to the description of tiger stripes (*sensu* Mugnai [19]). Brown et al. [68] replicated tiger stripes, however not to an extent statistically greater than non-inoculated plants. Only Ye et al. [62] recently replicated tiger stripe-like symptoms, in 25% of inoculated plants, using an isolate of *Fomitiporia punicata*. To our knowledge, this species has never been reported in vineyards outside of China.

Overall, these results partly fulfill Koch’s postulates, as several *Hymenochaetales* induced the appearance of wood symptoms, including white rot, but not tiger stripes in the leaves (with one exception; [62]). In most cases, wood symptoms were significantly larger than those found in control vines, nevertheless, several authors report low re-isolation rates, especially as regards Fmed [45,64,65,71,72], suggesting a possible involvement of cultural bias. High variability in the re-isolation rates of Fmed has also been documented in greenhouse essays with olive trees [73] and kiwifruit [74]. 

## 4. Insights from Recent Microbiome Studies

Culture-independent microbiome studies, such as those based on next-generation sequencing (NGS), may unveil the microbial ecology of endophytic communities present in the wood of esca-affected plants and their role in the development of leaf symptoms. Among the numerous points in favor of culture-independent methods, NGS studies allow insights over unculturable and rare taxa, largely overcoming the cultural methods bias, and provide reasonable estimates for microorganisms’ relative abundances [75]. Over the last three years, four NGS-based studies investigated the microbial ecology of esca proper-affected plants [76,77,78,79]. We hereby report some of the main findings and later discuss how they improve the state-of-the-art, over the involvement of white rot fungi in esca symptoms in leaves. 

In Portugal, Del Frari et al. [76] compared the fungal communities present in the perennial wood in the proximity of leaf-symptomatic canes to those of asymptomatic canes, using DNA metabarcoding. The authors reported an underrepresentation of genera *Cryptococcus*, *Ramularia*, *Debaryomyces*, *Cladosporium*, as well as Pch, in the perennial wood near symptomatic canes. The only genus overrepresented in this woody tissue was *Fomitiporia* sp. The authors suggested that the overrepresentation of this taxa may contribute to a higher wood decay activity (and therefore of byproducts of wood degradation), which may support the second theory of leaf symptoms’ manifestation [19].

In France, Bruez et al. [77] analyzed the endophytic microbiome of leaf-symptomatic vines and compared it to vines that never manifested esca symptoms in leaves. The authors report that *Aureobasidium pullulans* and Pch were more abundant in the cordons of asymptomatic plants. White rot was present in 70% of leaf-symptomatic plants, while it was absent in asymptomatic ones. Unsurprisingly, white rot tissue was associated with the presence of Fmed and a minor abundance of Pch, while non-necrotic tissue was dominated by Pch, lower percentages of Fmed and other pathogenic and non-pathogenic endophytes. The authors stated that “white rot-associated microbiota is essential for the onset of esca disease” [77]. They suggested that the interaction among Fmed, Pch and bacteria may lead to the production of phytotoxic secondary metabolites responsible for leaf symptoms’ appearance. This hypothesis reminds those proposed by Mugnai et al. [19].

In a second study, Bruez et al. [78] examined the effect of sodium arsenite on the endophytic mycobiome of leaf-symptomatic vines. Upon inspection, white rot was present in the stem of 60 to 100% of leaf-symptomatic plants, in three different vineyards. Remarkably, none of the sodium arsenite-treated plants exhibited tiger stripes in the following growing season, while 100% of non-treated vines manifested them, highlighting the effectiveness of the treatment. Non-treated plants revealed the presence of high abundances of Fmed in different tissue types. The microbial dynamics of the fungal microbiome of treated plants were profoundly altered by the treatment, highlighting a strong decrease in the abundance of three fungi (Fmed, *Seimatosporium vitis* and *Mycena maurella*). The greatest reduction concerned Fmed, and it was recorded in all tissue types examined. Along with the strong decrease in Fmed abundance, several fungi, including Pch, increased their own, revealing their higher tolerance to sodium arsenite when compared to Fmed. 

In Italy, Pacetti et al. [79] studied the microbiome of plants before and after trunk surgery treatment, a technique that consists in removing white rot by mechanical means. The authors reported leaf symptoms remission after fully removing white rot from previously symptomatic plants. Treated plants revealed a significant decrease in Fmed abundance, while leaving nearly unaffected Pch abundance. These interesting results led the authors to state that ‘the Fmed activity in wood colonization had a relevant role in foliar symptom expression in esca proper-affected vines’. However, the authors also suggest that some wood degradation mechanisms could be in common with agents of different wood symptoms and degradation [79]. 

## 5. What Did We Learn?

In consideration of the evidence provided in this article, we try to answer two main questions. (1) Is the ‘toxins hypothesis’ still the most credible? (2) Can we control leaf symptoms’ manifestation? 

### 5.1. Toxins Hypothesis

Examining, together, field data and artificial inoculations, we gather that neither the presence of white rot nor that of Fmed is sufficient to trigger the manifestation of tiger stripes. The same applies to Ascomycetes, such as Pch and Pmin, which are responsible for producing fungal toxins and other phytotoxic molecules [80], whom are rarely found alone (i.e., without the simultaneous presence of Fmed or associated symptoms) in leaf-symptomatic plants. Arguably, limitations such as cultural bias and individual approaches to wood sampling and fungal isolation may have hidden the presence of white rot fungi in such studies. On the contrary, Pch and/or Pmin seem to be often found, in similar abundances, in both leaf-symptomatic and asymptomatic plants (e.g., [41,55,76]). Altogether, this evidence supports the view that the presence of both Fmed and Pch or Pmin is necessary to trigger leaf symptoms’ manifestation. Another point in favor of this conclusion is represented by the situation in South America, New Zealand and, to a lesser extent, Australia and South Africa. In these geographical areas, Ascomycetes like Pch and *Phaoacremonium* spp. are often found in grapevine wood, even in high abundances [81], but tiger stripes in leaves are rarely reported, if reported at all. Coincidentally, Fmed has not been encountered affecting grapevines neither in South America nor in New Zealand, while other species of the *Fomitiporia* genus are present, but rather infrequent, in South Africa and Australia.

As suggested by Bruez et al. [78], competition-induced metabolites may be good candidates for explaining leaf symptoms. Hypothetically, environmental perturbations in the plant-fungi interaction dynamics (with a possible involvement of bacteria [77,79,82]) may trigger the release of such chemicals that reach the canopy and lead to tiger stripes. This scenario would explain, to some extent, the reasons behind annual symptoms discontinuity and the influence of abiotic factors such as climatic conditions (e.g., spring rain and/or heatwaves, [83,84,85,86,87]), as well as the difficulties in replicating tiger stripes by artificial inoculations. On the other hand, numerous studies consistently report a high abundance of Fmed and white rot in leaf-symptomatic plants, suggesting that byproducts of wood degradation (and/or chemicals such as volatile organic compounds; [88,89]), may play a more important role than currently thought. In fact, as previously illustrated, fighting Fmed with sodium arsenite or removing white rot by trunk surgery, successfully prevents leaf symptoms’ manifestation. While the ‘toxins hypothesis’ cannot be fully dismissed, it may be less relevant than currently believed, in favor of the ‘byproducts of wood degradation hypothesis’ or the most recent ‘competition-induced metabolites’ hypothesis. Nevertheless, they remain hypotheses and further research is needed.

### 5.2. Control of Leaf Symptoms

Treating vines with sodium arsenite successfully controlled esca for decades and, up until recently, its mode-of-action was unknown. Today, we know that this chemical affects the plants physiology and microbial ecology, successfully preventing leaf symptoms’ manifestation [15,78]. Similarly, trunk surgery, an ancient practice recently rediscovered, is capable of strongly reducing the expression of leaf symptoms for several years after treatment [18,79]. The common ground between these two different treatments lies in their drastic effect on Fmed (the former) and white rot (the latter). While it is not possible to exclude secondary effects not yet assessed, it is reasonable to assert that this fungus plays a significant role in the expression of leaf symptoms. In fact, field evaluations and, to a lesser extent, pathogenicity tests suggest that both the presence of white rot fungi and tracheomycotic Ascomycetes is necessary to trigger the expression of leaf symptoms. Besides, Pch does not seem to be significantly affected by sodium arsenite [78], and if this fungus alone was responsible for tiger stripes in leaves, it is hard to motivate how this treatment (or trunk surgery) effectively prevented symptom manifestation. The next question that naturally arises is: Can we control tiger stripes’ manifestation exclusively by targeting Fmed (or other white rot fungi)? Available evidence suggest that the answer may be **yes**, however, alternatives as effective as sodium arsenite are not yet available, and trunk surgery is an expensive and time-consuming technique not suitable for all vine growers. While chemical solutions are being developed, some of which showing promising results [16,17], preventing infections of Fmed by protecting fresh and old pruning wounds may be a solution worth investigating [23].

## 6. Conclusion and Perspectives

### 6.1. Esca Classification

Over the years, several authors have proposed classifications for ‘esca’, e.g., as multiples syndromes or a disease complex, in the effort of joining diverse internal and external symptoms along with numerous putative and confirmed fungal pathogens [9,39]. The evidence provided in this review suggests that the current classification into five separate syndromes [9] may require a re-evaluation. Firstly, field data does not support the existence of the syndrome called white rot (formerly ‘esca’; [39]). To the best of our knowledge, there are no reports of vineyards affected exclusively by this syndrome (absence of wood symptoms caused by trachemycotic fungi). Secondly, studies that describe GLSD are infrequent, and may have been subject to several biases. The presence of white rot is elusive, especially if vines are not thoroughly screened (e.g., area near pruning wounds), and Fmed may be in decaying wood before the appearance of white rot, as suggested by recent radiodensity measurements [90]. However, this hypothesis needs to be confirmed in future culture-independent studies, which employ accurate technologies for white rot detection (e.g., X-ray; [90]). Due to the elusiveness of internal symptoms, which can be assessed almost exclusively only destructively, defining all vines that exhibit or have exhibited, at least once, leaf symptoms as affected by ‘esca’, may simplify communication. In fact, it is often necessary to clarify, in colloquial speech and scientific writing, whether the term ‘esca’ is used to identify leaf-symptomatic plants (i.e., GLSD), vines affected by white rot or esca proper. This suggested classification of esca aims to join GLSD and esca proper into a single disease, i.e., ‘esca’, and it may be further discussed within the phytopathological community.

### 6.2. Leaf Symptoms

Nearly all the evidence provided in this review suggest that the simultaneous presence, and action, of tracheomycotic fungi and members of the *Hymenochaetales,* most frequently Fmed, is necessary to trigger esca symptoms in leaves. Removing one of the two factors from the equation, i.e., white rot fungi, prevents or at least delays leaf symptoms’ relapse. While fungal pathogens are a pre-requisite, numerous other factors have to be accounted for in the tiger-stripes equation, some of the most intriguing being the nutritional and physiological status of the plants [14] and the role of the *Botryosphaeriaceae* [91]. In this review, we propose an alternative view of esca symptoms in leaves, based on past etiological and epidemiological data and recent evidence; however, our effort remains confined to the field of microbiology and a literature analysis that has taken into account all other areas of leaf symptoms research may shed further light over this fascinating subject.

## Figures and Tables

**Table 1:** Artificial inoculation of *Hymenochaetales* in potted vines or in field studies and induction of wood and leaf symptomatology.

Fungal Isolates	Symptoms			Essay Length (Months)	Grapevine Cultivar	Potted Plants (P)/Field Study (F) Plant Age (Years Old)	Country/Reference
	White Rot	Tiger Stripes (%)	Other Wood Symptoms ^#^				
*Fomitiporia mediterranea*	*n*	*n*	y	6	Cabernet Sauvignon	*p* (1)	France [45]
				18			
*F. mediterranea* (10 isolates)	*n* *	*n*	y	15	Cabernet Sauvignon	*p* (1)	France [64]
*F. mediterranea* (3 Isolates)	*n*	*n*	y	9	Macabeo	*p* (1)	Spain [65]
			*n*		Tempranillo		
*Inocutis* sp.	*n*	*n*	y	15	Carménère	*p* (2)	Chile [66]
				2	Cabernet Sauvignon	F (1)	
*F. mediterranea*	*n*	*n*	y	4	Sultana Seedless	*p* (1)	Turkey [67]
*F. polymorpha*, *F. langloisii* and *Tropicoporus texanus*	*n*	y ^+^	y	12	Crimson Seedless	*p* (1)	United States [68]
*F. mediterranea*	*n*	*n*	y	10	Kolahdari	*p* (2)	Iran [69]
*F. punicata*	*n*	25%	y	3	Cabernet Franc	*p* (2)	China [62]
*F. mediterranea*	y	*n*	y	96	Emperor	F (7)	United States [70]
*F. mediterranea* (3 isolates)	y	*n*	*n*	24	Sangiovese	F (13)	Italy [59]
	y **		y	8	Italia	F (6)	
					Matilde	F (9)	
*F. mediterranea*	y	11.1–22.2% ^++^	y	36	Italia	F (5)	Italy [71]
		*n*			Matilde	F (9)	
*Inonotus setuloso-croceus*, *Fomitiporella* sp., *F. capensis*, *Phellinus* sp., others	y	*n*	*n*	24	Shiraz	F (10)	South Africa [68]
			Y º		Mourvédre		

‘*n*’ indicates the absence and ‘y’ the presence of specific wood or leaf symptoms. (^#^) Wood symptoms belong to the following categories: discoloration, brown streaking, brown lesions, necrosis. (*) The authors report the presence of white mycelium and ‘lesions that looked like white rot’ [64]. (**) described as ‘first signs of spongy wood decay’ [59]. (^+^) Symptoms resembling tiger stripes were observed for all four isolates, however their frequency of appearance was not significantly different from non-inoculated control plants. (^++^) Described as ‘chlorosis and reddening of the leaf margins; necrosis of large parts of the lamina’ [71]. (º) Only for isolates *I. setuloso-croceus* and *Fomitiporella* sp.

## Data Availability

Not applicable.
